# Multidimension Analysis of the Prognostic Value, Immune Regulatory Function, and ceRNA Network of LY6E in Individuals with Colorectal Cancer

**DOI:** 10.1155/2022/5164265

**Published:** 2022-03-11

**Authors:** Ting Li, Weidong Liu, Chun Wang, Man Wang, Wenjia Hui, Jiajie Lu, Feng Gao

**Affiliations:** ^1^Department of Gastroenterology, People's Hospital of Xinjiang Uygur Autonomous Region, China; ^2^Xinjiang Clinical Research Center for Digestive Diseases, China; ^3^Department of Pathology, People's Hospital of Xinjiang Uygur Autonomous Region, China

## Abstract

**Background:**

Lymphocyte antigen 6 complex, locus E (*LY6E*) is abnormally expressed in several cancers and is associated with poor outcomes. However, the biological role of *LY6E* in colorectal cancer (CRC) remains largely unknown. Hence, we aimed to evaluate the expression levels, prognostic value, biological functions, and immune effects of *LY6E* via pan-cancer and CRC analyses using multiple databases.

**Methods:**

We analyzed the expression pattern of *LY6E* in various cancers. The prognostic value of *LY6E* expression was identified using the Kaplan–Meier analysis and the Cox regression models. We used gene set enrichment analysis (GSEA) to identify the potential functions of *LY6E*. Correlations between the *LY6E* expression and various factors, including *LY6E* methylation level, copy number variation (CNV), microsatellite instability (MSI), and immune checkpoint genes, were also analyzed. The levels of *LY6E* expression and immune infiltration were analyzed using CIBERSORT. We constructed a regulatory network that was in compliance with the competing endogenous RNA (ceRNA) hypothesis. A ceRNA expression-based nomogram was established. Real-time PCR (qRT-PCR) was applied to validate the expression of *LY6E*-related ceRNA in CRC cell lines.

**Results:**

*LY6E* is overexpressed in several tumor types, including CRC, and patients with high expression levels of *LY6E* have a poor prognosis. The Kaplan–Meier analysis and Cox regression analysis showed that *LY6E* could be considered a favorable prognostic factor in TCGA and GEO cohort. The results of GSEA showed that high *LY6E* expression levels were associated with immune-related pathways, such as those involved in antigen processing and presentation and the intestinal immune network for IgA production. Six methylation sites of *LY6E* that were associated with prognostic survival were screened. Moreover, the high levels of *LY6E* expression were correlated with copy number gain, microsatellite instability high, and immunotherapy response. The results of CIBERSORT analysis demonstrated that the *LY6E* expression levels were correlated with the infiltration of multiple immune cells, especially T cells. Then, we constructed a ceRNA network (LINC00963/miR-92a-3p/*LY6E*) and validated it using qRT-PCR. A predictive ceRNA-based nomogram was established and validated.

**Conclusion:**

The oncogenic *LY6E* may serve as a promising marker for the diagnosis and treatment of CRC.

## 1. Introduction

Globally, colorectal cancer is the third most frequently diagnosed malignancy and the second leading cause of cancer-related mortality among people [1]. It is characterized by a high degree of malignancy and invasiveness, poor prognosis, and a high mortality rate. In 2018, colorectal cancer was estimated to account for 1.8 million new cases of cancer and 881,000 deaths worldwide [2]. Tumor recurrence and liver metastasis are still majorly attributed to the high mortality of CRC patients [3]. While considerable progress has been made in the development of therapeutic strategies, in most patients, cancer remains undetected until it progresses to an advanced stage, and the 5-year survival rate is merely 14% [4]. Early diagnosis and resection may effectively improve patient outcomes. Hence, it is necessary to discover meaningful prognostic markers that improve oncological outcome prediction in CRC patients.

Lymphocyte antigen 6 complex, locus E (*LY6E*) is a member of the lymphocyte antigen-6/urokinase-type plasminogen activator receptor Ly6/uPAR family, also referred to as thymic shared antigen-1 (TSA-1) or stem cell antigen-2 (SCA-2) [5]. Importantly, the biological functions of *LY6E* have been linked to viral infections [6], immune regulation [7], and tumor metastases [8]. *LY6E* exhibits transcriptional activity in numerous tissues in organs such as the lung, liver, uterus, spleen, and ovary [9]. It was found that *LY6E* was overexpressed in a multitude of tumor types, including breast, gastric, esophageal, and colorectal tumors, which implies that it might be involved in tumorigenesis and tumor progression [10]. Furthermore, a high level of expression of *LY6E* is strongly associated with a poor prognosis [11]. Thus, *LY6E* expression might represent a promising prognostic biomarker, but its biological functions in CRC are yet to be elucidated thoroughly.

In recent years, immune checkpoint blockade therapy (ICBT) has made tremendous breakthroughs in oncology treatment and has demonstrated remarkable therapeutic prospects for recurrent or metastasized cancer. For CRC patients, ICBT has also displayed promising efficacy and tolerability in clinical trials, providing a novel treatment for patients with advanced or drug-resistant CRC. However, there are significant differences in response to immunotherapy among patients with different types of CRC. ICBT is highly effective in microsatellite instable (MSI) patients with CRC but not in microsatellite stable (MSS) [12, 13]. A possible explanation is that CRC with MSI may have a higher tumor mutational burden (TMB) [14]. The density of neoantigens has also been reported to affect the efficacy of ICBT [15, 16]. Hence, not all patients would benefit from ICBT. Predictive biomarkers to predict prognosis and treatment response are therefore required for guiding personalized treatment and improving clinical outcomes.

Noncoding RNAs (ncRNAs) have attracted considerable attention from researchers, as they play essential roles in tumor growth, progression, and metastasis. MicroRNA (miRNA) and long noncoding RNA (lncRNA) are well-studied species of small ncRNAs [17, 18]. It was found that the inhibition of miRNA on mRNA may be partially inhibited if lncRNA and mRNA share the same miRNA recognition elements [19]. Over recent years, a certain ceRNA pattern has been widely observed in the latest studies on CRC [20]. Accordingly, this has been used for the development of a key and novel player in cancer therapy [21, 22]. Few studies have reported on the relationship between *LY6E* and the ceRNA regulatory network in individuals with CRC.

In this study, we analyzed the differences in *LY6E* expression in colorectal cancer and other types of cancer. The relative association between *LY6E* expression and immune cell infiltration was evaluated, and the functions, prognostic value, methylation sites, copy number variation (CNV), microsatellite instability (MSI), immune checkpoint genes, and regulatory ceRNA network associated with *LY6E* were assessed. The presence of *LY6E* and *LY6E*-associated ceRNA expression was confirmed via qRT-PCR in colon cancer cells. In summary, this is the first study to elaborate upon the underlying mechanisms of *LY6E* in CRC by performing a multidimensional analysis.

## 2. Materials and Methods

### 2.1. TCGA, GTEx, and GEO Data

The HTSeq-FPKM transcriptome data, clinical data, DNA methylation data, CNV, MSI status, and miRNA data of Colon adenocarcinoma (COAD) were downloaded from TCGA data portal (https://tcga-data.nci.nih.gov/tcga). Only samples for which clinical survival information was available were selected for analysis. However, because of the unavailability of an adequate number of colonic samples for control groups in TCGA database, we downloaded 308 text files containing the results obtained after the analysis of healthy colon samples from the GTEx portal (https://www.gtexportal.org/home/datasets). The data obtained from the two datasets were merged into one large combined dataset that was used to compare the levels of LY6E in the controls and CRC specimens. Additionally, we utilized TCGA data to analyze the expression of LY6E in other cancer types. Data for GSE17538 and GSE38832 were downloaded from the Gene Expression Omnibus database (GEO, http://http://www.ncbi.nlm.nih.gov/geo) and used to validate the prognostic value of LY6E in CRC patients.

### 2.2. Prognostic Predictive Value of *LY6E*

Control samples and samples obtained from patients with a survival duration of less than 30 days were excluded before survival analysis. Patient samples were sorted by the levels of expression of *LY6E* as samples exhibiting the lowest to highest expression levels, and the optimal cutoff point for *LY6E* expression was determined using the “survminer (version 0.4.9)” R package. Progress-free survival (PFS) analysis and overall survival (OS) analysis of *LY6E* were performed using the “survival (version 3.2-13)” package of R software (version 4.1.2). Additionally, we determined the prognostic value of *LY6E* in the GSE17538 and GSE38832 datasets using the optimal cutoff point. We then used univariate and multivariate Cox risk regression analyses to identify whether *LY6E* expression levels and the corresponding clinical-pathological features were independent prognostic factors. We extended our analysis to correlation between *LY6E* expression with CNV, MSI, and several well-known immune checkpoint genes. TCGA divided MSI into three types: MSS (microsatellite stable), MSI-L (microsatellite instability low), and MSI-H (microsatellite instability high) in its clinical data. If *P* < 0.05, values were considered significant.

### 2.3. Gene Set Enrichment Analysis

Gene set enrichment analysis (GSEA) software version 4.1.0 was used to analyze the potential function of *LY6E* with a permutation of 1000. Patients were categorized into the “high” and “low” groups based on the optimal cutoff value for *LY6E* expression. Kyoto Encyclopedia of Genes and Genomes (KEGG) analysis (c2.cp.kegg.v7.4.symbols.gmt) was performed to identify the crucial pathways involved in the groups exhibiting low versus high levels of *LY6E* gene expression. As recommended by the GSEA User Guide (https://www.gsea-msigdb.org/gsea/doc/GSEAUserGuideFrame.html?Interpreting_GSEA), a false discovery rate (FDR) of <0.25 and a nominal *P* value of <0.05 indicated that differences were statistically significant.

### 2.4. Analysis of CRC Survival-Related CpG Sites of *LY6E*

The DNA methylation levels derived from TCGA datasets were expressed as *β* values. We identified and visualized the methylation sites in the *LY6E* promoter region. The Pearson correlation analysis was used to assess the correlation between *LY6E* expression and the mean methylation level at *LY6E* CpG sites and the methylation at each promoter CpG site. Finally, we performed the Kaplan–Meier analysis and used a Log-rank test to determine whether methylation levels at the *LY6E* promoter CpG sites were associated with OS in CRC patients. If the *P* value was <0.05, values were considered statistically significant.

### 2.5. Prediction of Prognostic ceRNA Network

The target miRNAs for *LY6E* were predicted using the ENCORI (http://starbase.sysu.edu.cn/) website [23]. In addition, we selected miRNAs that were more eligible for the construction of the ceRNA network by analyzing the correlation between miRNA expression and *LY6E* expression. Then, specific survival-related miRNAs were screened and utilized to generate ceRNA networks. The target lncRNAs of miRNAs were also predicted using the ENCORI website, and lncRNAs that were negatively correlated with the expression of selected miRNAs were filtered out. Specific lncRNAs that were correlated with survival were used for ceRNA network construction.

### 2.6. Correlation of *LY6E* Expression with Immune Cell Infiltration

To understand the effect of *LY6E* on the immune response, we calculated the relative proportions of 22 infiltrating immune cells using the CIBERSORT algorithm [24]. The CIBERSORT algorithm is used for the analysis of gene expression profiles of complex tissues to determine their cellular composition. Next, the Wilcoxon rank-test was performed to identify changed significantly immune cells between the groups exhibiting high and low *LY6E* expression. Finally, the correlation between *LY6E* expression levels and immune cell infiltration was analyzed using the Pearson correlation test. A *P* value < 0.05 was considered statistically significant.

### 2.7. Development of the Nomogram

A nomogram was used to predict CRC prognosis. The nomogram was developed using the “rms (version 6.2-0)” package of R software and included an analysis of *LY6E*-related ceRNA expression levels and corresponding clinicopathological features. We then plotted the calibration graph to evaluate the differences between the observed and nomogram-predicted OS.

### 2.8. Cell Lines and qRT-PCR

The colon epithelial cell line NCM460 (BNCC339288) and colorectal cancer cell line SW480 (CL-0223) were used to validate the expression. The cells were cultured in DMEM (Gibco) medium containing 10% fetal bovine serum (Biological Industries) in a humidified incubator at 37°C and 5% CO_2_. The total RNA in the cell lines was extracted using the TRIzol reagent. Then, cDNA was synthesized using a RevertAid First Strand cDNA Synthesis Kit (K1622, Thermo Fermentas). qRT-PCR was performed using the iQ5 cycler system (BioRad). Statistical analyses were performed using GraphPad Prism 8.0.1 software. The expression levels of *LY6E*, hsa-miR-miR-92a-3p, and LINC00963 were analyzed using the *t*-test; if *P* ≤ 0.05, values were considered significant. Primers used for qRT-PCR are listed in Table 1.

## 3. Results and Discussion

### 3.1. *LY6E* Expression Levels in Different Types of Tumors

**Figure 1:**
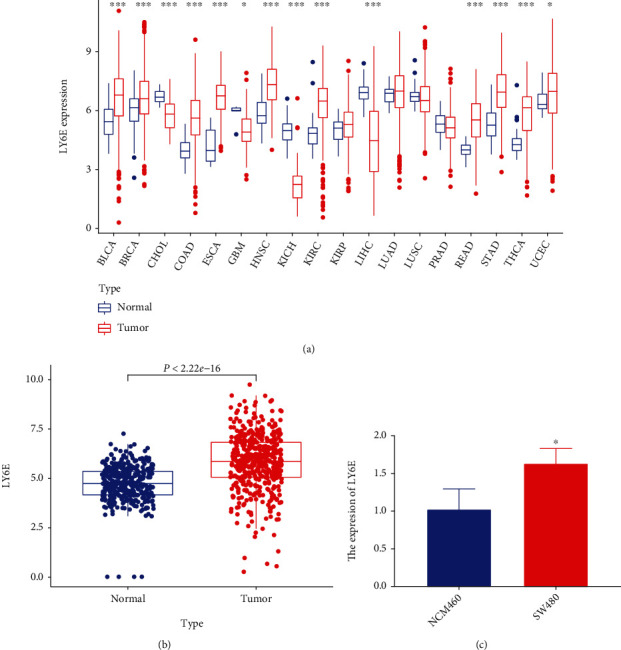
LY6E expression levels in pan-carcinoma and colorectal cancer (CRC). (a) TCGA cohort analysis of the mRNA expression of LY6E between normal and tumor tissues. (b) TCGA combined GTEx database shows that LY6E is upregulated in CRC. (c) The expression of LY6E in human normal colon epithelial cell NCM460 and human colorectal cancer cell line SW480.

We compared the *LY6E* expression levels in both cancerous and healthy tissues. The results revealed that higher levels of *LY6E* were expressed in individuals with breast invasive carcinoma (BRCA), bladder urothelial carcinoma (BLCA), esophageal carcinoma (ESCA), head and neck squamous cell carcinoma (HNSC), kidney renal clear cell carcinoma (KIRC), liver hepatocellular carcinoma (LIHC), skin cutaneous melanoma (SKCM), stomach adenocarcinoma (STAD), and thyroid carcinoma (THCA). A converse expression pattern was observed in individuals with cholangiocarcinoma (CHOL), glioblastoma multiforme (GBM), kidney chromophobe (KICH), liver hepatocellular carcinoma (LIHC), lung squamous cell carcinoma (LUSC), and prostate adenocarcinoma (PRAD) (Figure 1(a)). An analysis of TCGA and GTEx datasets showed that *LY6E* expression levels were higher in CRC patients (Figure 1(b)). The qRT-PCR results showed that *LY6E* expression was significantly higher in the CRC cell line SW480 than in healthy human colonic epithelial cells from the NCM460 cell line (Figure 1(c)).

### 3.2. Prognostic Value of *LY6E* in CRC Patients

**Figure 2:**
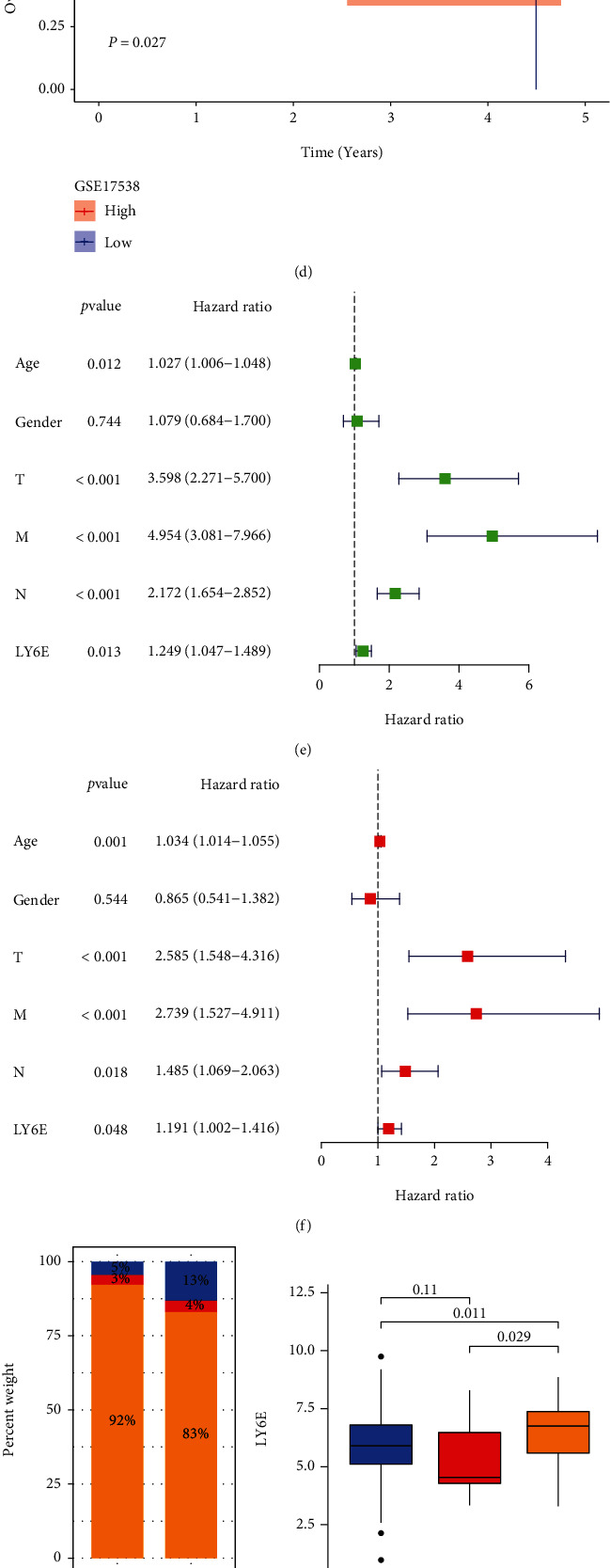
Prognostic predictive value of LY6E. (a) The Kaplan–Meier curve showed that high LY6E mRNA expression is significantly associated with poor overall survival and (b) progress-free survival compared with low expression. (c) The GSE17538 and (d) GSE38832 datasets show that patients with high LY6E expression were closely correlated with poorer overall survival. (e) Univariate and (f) multivariate Cox regression analyses estimating prognostic value of LY6E. (g) The rate of copy number gain and (h) MSI-H was higher in the LY6E high-expression group. (i) The mRNA expression of LY6E is positively correlated with immune checkpoint molecules expression level.

The optimal cutoff value for *LY6E* expression was determined to be 7.306 using the survminer package. We categorized the patients into the high and low-expression groups using this optimal cutoff value. The Kaplan–Meier curve showed that the overexpression of *LY6E* was correlated with a poorer prognosis (Figure 2(a)). This result was further validated with the data for GSE17538 and GSE38832 (Figures 2(c) and 2(d)). Further analysis revealed that the high expression levels of *LY6E* were significantly positively correlated to a worse PFS (Figure 2(b)). Cox regression analysis was used to identify whether *LY6E* expression was an independent prognostic factor. The univariate Cox model revealed that *LY6E* expression levels were associated with OS (HR = 1.191, *P* = 0.048, 95% CI [1.02-1.416]) (Figure 2(e)). Subsequently, the results of multiple Cox regression analyses demonstrated that the expression levels of *LY6E* remained a risk factor for OS (HR = 1.191, *P* = 0.048, 95% CI [1.002-1.416]) (Figure 2(f)).

CNV of *LY6E* was also analyzed; the results indicated the rate of copy number gain was higher in the *LY6E* high-expression group (Figure 2(g)). The same tendency was maintained regarding the percentage change in MSI-H (Figure 2(h)). We then analyzed the relationship between *LY6E* and several immune checkpoint genes. A positive correlation was found between *LY6E* with PD-1, LAG-3, VISTA, TIM-3, IDO1, PD-L1, TIGIT, and CTLA-4 expressions (Figure 2(i)). Taken together, the results suggest that the abnormal levels of expression of *LY6E* were a potential prognostic factor and predicted the clinical benefit for the immune checkpoint blockade for CRC patients.

### 3.3. GSEA

**Figure 3:**
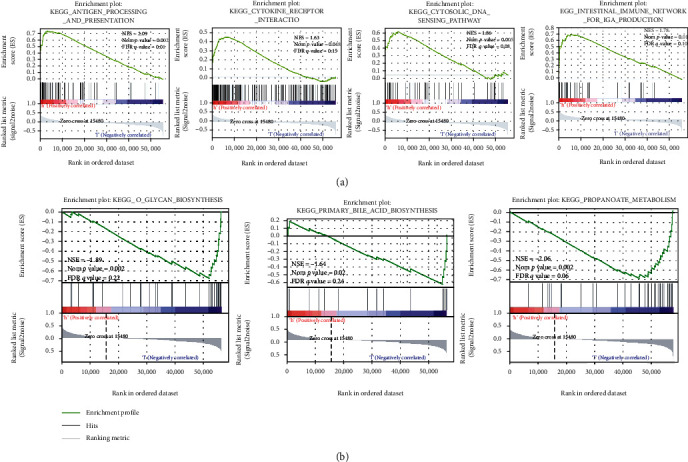
GSEA. (a) The major enriched pathways for the high-expression group. (b) The primary enriched pathways for the low-expression group.

GSEA is a computational approach for the detection of minor undetectable changes in gene expression. GSEA results indicate that the group exhibiting a high level of *LY6E* expression was mainly enriched in antigen processing and presentation, the intestinal immune network for IgA production, and natural killer cell-mediated cytotoxicity (Figure 3(a)). The primary enriched pathways for the low-expression group included the pathways for propanoate metabolism, o-glycan biosynthesis, and primary bile acid biosynthesis (Figure 3(b)).

### 3.4. Analysis of CRC Survival-Related CpG Sites of *LY6E*

**Figure 4:**
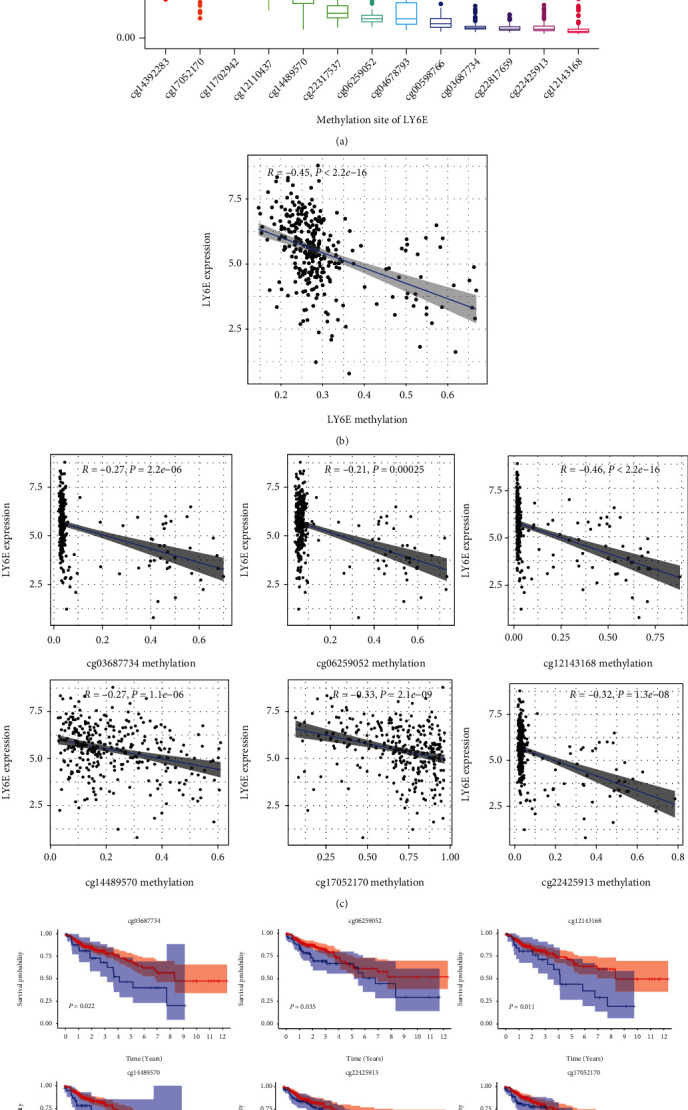
CRC survival-related CpG sites of LY6E. (a) Thirteen CpG sites in the promoter region of LY6E. (b) LY6E expression was negatively correlated with the mean methylation level of LY6E. (c) LY6E expression was negatively correlated with the methylation level at each promoter CpG site. (d) Six of these methylation sites were linked to a poorer overall survival.

Because hypermethylation and hypomethylation are associated with the downregulation and upregulation of genes, respectively, we examined the methylation status along with the *LY6E* gene locus of CRC samples and healthy colonic tissue samples obtained from TCGA database (Figure 4(a)). Thirteen CpG sites were identified in the promoter region of *LY6E*, and the methylation levels at these different sites are shown in Figure 4(a). Six of these methylation sites were found to be significantly linked to a poorer OS (*P* < 0.05) (Figure 4(d)). Meanwhile, *LY6E* expression was found to be negatively correlated with the mean methylation level at these sites (Figures 4(b) and 4(c)).

### 3.5. Correlation between *LY6E* Levels and Immune Cell Abundance in CRC

**Figure 5:**
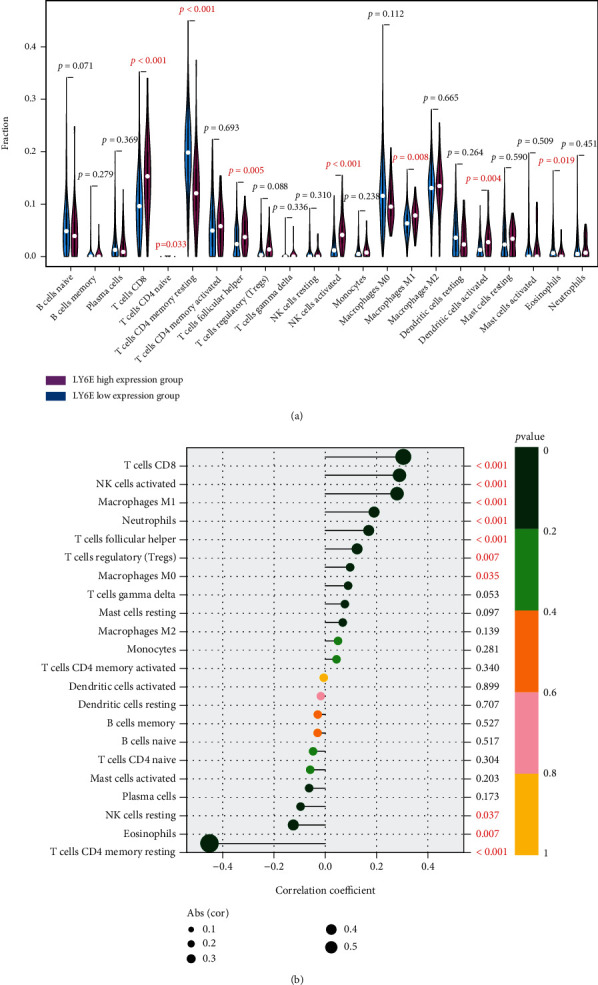
Correlations of LY6E expression with immune infiltration level in CRC. (a) The change ratio of immune cell types in the high LY6E- and low LY6E-expression groups in colorectal cancer samples. (b) LY6E expression was positively correlated with the infiltration level of CD8+ T cells and negatively correlated with CD4 memory resting T cells.

CIBERSORT analysis was performed to determine whether *LY6E* expression was correlated with the level of immune infiltration in CRC patients. We categorized the patients into the high *LY6E*- and low *LY6E*-expression groups based on optimal cutoff values. The results of our analysis revealed that the group exhibiting a higher expression of *LY6E* had a higher abundance of CD8+ T cells (*P* < 0.001), follicular helper T cells (*P* = 0.005), activated NK cells (*P* < 0.001), M1 macrophages (*P* = 0.008), activated dendritic cells (*P* = 0.004), and a relatively low proportion of resting memory CD4+ T cells (*P* = 0.004) and eosinophils (*P* = 0.019) (Figure 5(a)). The results of correlation analysis suggested that *LY6E* expression was positively correlated with the infiltration level of CD8+ T cells (*r* = 0.30, *P* < 0.001) and negatively correlated with that of CD4 memory resting T cells (*r* = −0.45, *P* < 0.001) (Figure 5(b)).

### 3.6. *LY6E*-Related ceRNA Network Construction in CRC

**Figure 6:**
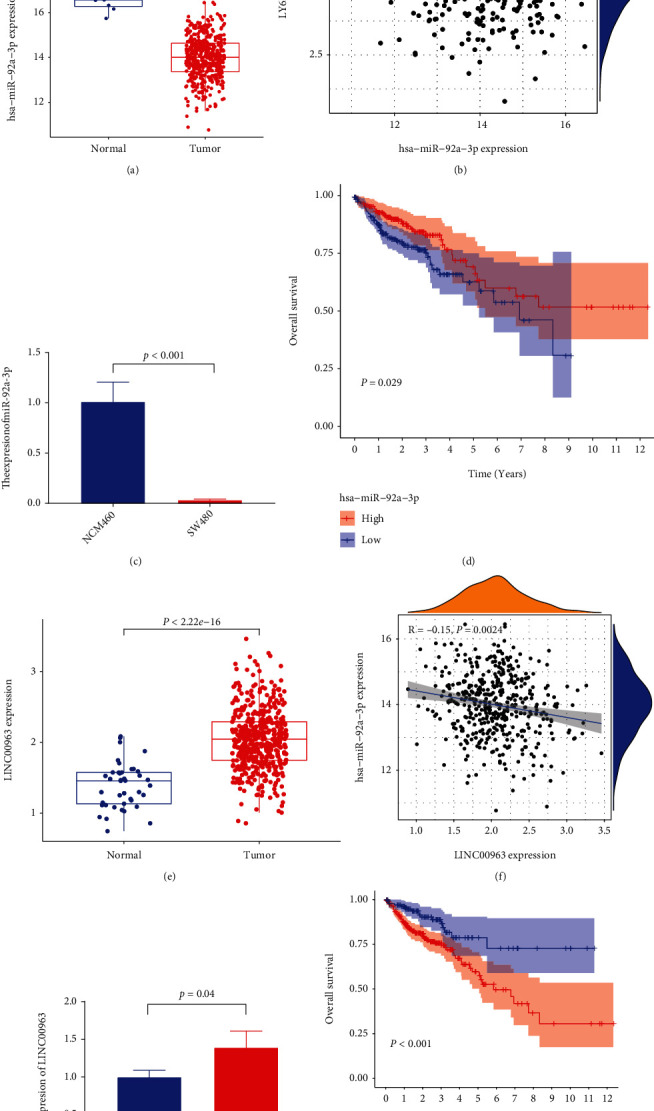
LY6E-related ceRNA network construction in CRC. (a) miR-92a-3p, a possible target miRNA for LY6E through the prediction of ENCORI database, is downexpressed in CRC from TCGA cohort. (b) miR-92a-3p expression was negatively correlated with LY6E expression. (c) The expression of miR-92a-3p in human normal colon epithelial cell NCM460 and human colorectal cancer cell line SW480. (d) Patients with low miR-92a-3p expression were closely correlated with poorer overall survival. (e) LINC00963, a possible target lncRNA for miR-92a-3p through the prediction of ENCORI database, is highly expressed in CRC from TCGA cohort. (f) LINC00963 expression was negatively correlated with miR-92a-3p expression. (g) The expression of LINC00963 in human normal colon epithelial cell NCM460 and human colorectal cancer cell SW480. (h) Patients with high LINC00963 expression were closely correlated with poorer overall survival.

**Figure 7:**
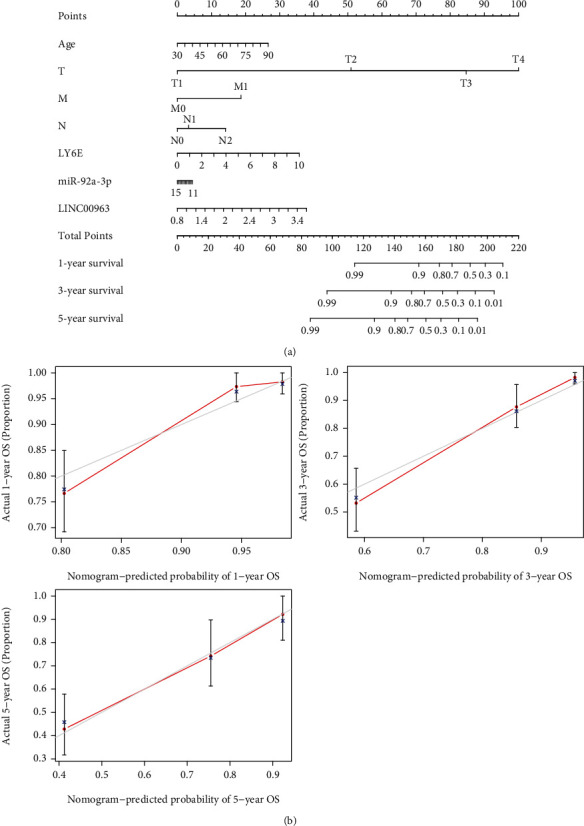
Development of the nomogram. (a) Nomogram using ceRNA expression levels and corresponding clinicopathological features for predicting the proportion of OS in CRC patients. (b) Calibration curve for predicting patient survival at 1 year, 3 years, and 5 years.

Predicted target genes from the ENCORI database were examined during correlation analysis. The expression levels of 12 miRNAs (hsa-miR-874-3p, hsa-miR-532-3p, hsa-miR-92a-3p, hsa-miR-339-5p, hsa-miR-497-5p, hsa-miR-520a-3p, hsa-miR-491-5p, hsa-miR-499a-5p, hsa-miR-361-3p, hsa-miR-204-5p, hsa-miR-15b-5p, and hsa-miR-383-5p) were negatively correlated with *LY6E* (Figures 6(a) and 6(b)). Survival analysis indicates a poorer prognosis in individuals with a low level of expression of miR-92a-3p (Figure 6(d)). We also utilized the ENCORI database to predict the binding of lncRNAs to hsa-miR-92a-3p. Based on the ceRNA network, we postulated that a negative correlation exists between lncRNAs and miRNAs. Correlation analysis showed that a target lncRNA expression level was negatively correlated with hsa-miR-92a-3p (Figures 6(e) and 6(f)). Survival analysis showed a greater association between the overexpression of LINC00963 and a poorer prognosis (Figure 6(h)). qRT-PCR results showed that the miR-92a-3p expression level was significantly higher in the NCM460 cell line than in healthy cells from the human colonic epithelial cell line SW480, while LINC00963 showed an inverse expression pattern in both the CRC cell lines (Figures 6(c) and 6(g)). A nomogram with ceRNA expression levels and corresponding clinicopathological features was generated (Figure 7(a)). The calibration plots showed good consistency between prediction by nomogram and observed results (Figure 7(b)).

## 4. Discussion

The important roles of the human Ly6 gene family were unappreciated until recently. To date, 20 human Ly6 proteins have been categorized and identified as either secretory or transmembrane proteins. *LY6E* could represent a promising candidate for antibody-drug conjugation development [25, 26]. In this study, we found that *LY6E* expression was upregulated in ten types of cancers using bioinformatic analysis and biologic validation, and these results are in accordance with those of previous studies [27, 28]. After combining the data obtained from the GTEx and TCGA database, *LY6E* expression was found to be significantly increased in CRC tissues compared to healthy tissues. An analysis of data from both TCGA and GEO databases confirmed that CRC patients with high *LY6E* expression levels exhibited a significantly poorer overall survival. Furthermore, the abnormal expression levels of *LY6E* were an independent prognostic factor that was also associated with CNV, MSI, immunotherapy response, and T cell infiltration. In summary, *LY6E* can be used as a prognostic biomarker for the diagnosis and treatment of CRC patients. Clinical trials of combination drugs against these targets have been designed. Previously, an antibody-drug conjugate directed against *LY6E* exhibited a powerful ability to kill tumors in preclinical models [25]. Subsequently, researchers developed a second-generation therapeutic that targeted *LY6E* and facilitated the improvement of therapeutic efficacy using additional cytotoxins [29]. Based on these encouraging results, we postulate that *LY6E* could become a promising agent for the treatment of CRC in the future.

The significance of *LY6E* in tumor growth is attributable to the fact that it might participate in immune regulation, especially for the modulation of T cell activation and proliferation [30, 31]. Wang et al. found that high levels of expression of LY6A/E in colonic epithelial cells from *E*. *faecalis-*colonized mice might lead to oncogenic transformation [32]. The increased expression of *LY6E* may promote tumorigenesis by affecting the TGF-*β* signaling pathway, INF-*γ* signaling pathway [8], and PTEN/PI3K/Akt/HIF-1 axis [33]. GSEA results suggested that in the group exhibiting high *LY6E* expression levels, *LY6E* was predominantly involved in the pathways associated with immune regulation. The above results suggest that *LY6E* may have multiple biological functions in tumors.

The role of the human LY6 gene in immune cell differentiation has not been understood thoroughly. Recently, Heng and Painter reported that mouse *LY6E* was expressed in activated T cells, immature T cells, thymus stromal cells, peripheral B cells, and macrophages, based on an analysis of the ImmGen database [34]. We used the CIBERSORT algorithm for immune cell quantization in different *LY6E* expression patterns. The results of this analysis suggested that the group exhibiting high levels of *LY6E* expression had a higher proportion of CD8+ T cells and a relatively lower proportion of resting memory CD4+ T cells. Moreover, we found that *LY6E* expression was significantly correlated with the number of CD8+ T cells and negatively correlated with the number of resting CD4+ T cells. These findings suggest that *LY6E* plays an important role in the regulation of immune infiltrating cells in CRC. The findings of similar previously performed studies have demonstrated that the *LY6E* peptide had the capability to provoke an immune response [35], specifically in modulating T cell activation and proliferation [30, 36, 37]. Another research shows that *LY6E*-loaded dendritic cells did efficiently stimulate T cell subtypes, and a significantly increased proportion of Th1 cells [35]. Thus, *LY6E* is an important molecule that can be used to target tumor antigens in multiple cancers.

The regulation of *LY6E* expression is fine-tuned by multiple growth factors and nuclear receptors [11]. DNA methylation acts as another common mechanism for its regulation. Recently, the experimental validation of the SNP242 C allele or methylation of the CpG site was associated with a reduced level of expression of LY6K [28]. However, there have been no reports of methylation studies on *LY6E*. We have been the first to screen five loci associated with prognosis. Moreover, we verified that the higher the level of methylation at these five loci, the better the prognosis of patients with CRC, suggesting that these methylated sites act as effective biomarkers for the prognosis of CRC patients. However, this needs to be confirmed by conducting additional experimental investigations.

According to ceRNA theory, lncRNAs could interact with mRNAs by competing with corresponding miRNAs in order to function as ceRNAs, to regulate mRNA expression [38]. We predicted the target miRNAs of *LY6E*. miR-92a-3p was found to be negatively correlated with *LY6E* expression and had a prognostic value for patients with CRC. Subsequently, we screened the upstream lncRNA of miR-92a-3p using the same process. Louis et al. found that circ-0000284 could function as a ceRNA of miR-637 to improve the expression of *LY6E* in individuals with cholangiocarcinoma [39]. Hu et al. found that the aberrant expression of miR-92a-3p derived from exosomes in the serum of CRC patients was strongly associated with tumor metastasis and chemotherapy resistance [40]. Lv et al. also revealed LINC00963 was upregulated in CRC tissues and promoted the proliferation, migration, and invasion of CRC cells by targeting miR-1281 [41]. Another study has reported that elevated LINC00963 expression levels are often indicative of a poor CRC prognosis [42]. These results proved the feasibility of our analysis to some extent. We also acknowledge that although the ceRNA network of *LY6E* was derived via bioinformatics analysis, further experimental verification is needed.

## 5. Conclusions

In conclusion, this is an initial analysis of the association between *LY6E* expression and methylation sites, CNV, microsatellite instability, immune checkpoint genes, and ceRNA networks in individuals with CRC. The modification of the methylation status within the gene promoter regulates the expression levels of *LY6E*. Different *LY6E* expression levels affect the immune cell infiltration level. Consequently, *LY6E* participates in tumorigenesis and progression. As an oncogenic protein, *LY6E* may serve as a promising marker for the diagnosis and treatment of CRC.

## Figures and Tables

**Table 1:** Primer sequences.

Name		Primer sequence
U6	Forward	5′-CTCGCTTCGGCAGCACA-3′
	Reverse	5′-AACGCTTCACGAATTTGCGT-3′
has-miR-92a-3p	Forward	5′-TTTCTACACAGGTTGGGGATCGGT-3′
	Reverse	5′-CGCAGGGTCCGAGGTATTC-3′
LY6E	Forward	5′-CATGGACATGCTGACAGGGT-3′
	Reverse	5′-TACAGGGACTGAGGCTCTCC-3′
LINC00963	Forward	5′-GTCAGGCCACTCTGCTACTG-3′
	Reverse	5′-CAACTGCGATGGTTGTGCTC-3′
*β*-Actin (human)	Forward	5′-CTCCATCCTGGCCTCGCTGT-3′
	Reverse	5′-GCTGTCACCTTCACCGTTCC-3′

## Data Availability

The data of this study are available in TCGA (https://portal.gdc.cancer.gov/), GTEx (https://www.gtexportal.org/home/datasets), and GEO database (https://www.ncbi.nlm.nih.gov/geo/).
